# In Vitro Research Tools in the Field of Human Immediate Drug Hypersensitivity and Their Present Use in Small Animal Veterinary Medicine

**DOI:** 10.3390/vetsci4010001

**Published:** 2016-12-22

**Authors:** Lavergne S. Lavergne

**Affiliations:** Department of Veterinary Clinical Medicine, College of Veterinary Medicine, University of Illinois–Urbana-Champaign, 2001 South Lincoln Av, Urbana, IL 61802, USA; sidolavergne@hotmail.com

**Keywords:** drug allergy, anaphylaxis, biomarkers

## Abstract

Drug hypersensitivity reactions (DHR) are immune-mediated idiosyncratic adverse drug events. Type I DHR are often referred to as “immediate” and involve B lymphocyte-secreted IgE that bind to the membrane of basophils and mast cells, inducing their degranulation. This review presents various in vitro tests that were developed in the field of human type I HS and implemented as clinical diagnostic tools in human cases of immediate DHR. The respective strengths and weaknesses of each test will be discussed in parallel of validation data such as specificity and sensitivity whenever available. Some of them have also been used as diagnostic tools in veterinary medicine, but not in cases of immediate DHR. Most of these diagnostic tools can be categorized into humoral and cellular tests. The former tests measure serum concentrations of factors, such as histamine, tryptase, and drug-specific IgE. The latter assays quantify markers of drug-induced basophil activation or drug-specific lymphocyte proliferation. Pharmacogenetic markers have also been investigated in immediate DHR, but not as extensively as in non-immediate ones. Throughout, practical aspects and limitations of the tests, as well as sensitivity and specificity parameters, will be presented. In addition, the experience of veterinary medicine with these diagnostic tools will be summarized. However, to date, none of them has ever been reported in a veterinary case of type I DHR.

## 1. Introduction

Approximately 20%–30% of adverse drug reactions are not directly related to the drug’s chemical or pharmacological properties and will only affect certain individuals. These reactions have sometimes been referred to as “Type B” reactions, with “B” referring to “bizarre” [1,2,3,4]. Some of these idiosyncratic reactions are related to genetic factors that predispose the patient to a direct drug toxic effect that other individuals will not experience: e.g., glucose-6-phosphate dehydrogenase deficiency and primaquine-associated hemolysis in humans; MDR1 mutation and ivermectin neurotoxicity in certain dogs. In other idiosyncratic drug reactions, the clinical signs are the consequence of the drug inducing a pathological immune reaction. These immune-mediated idiosyncratic drug events have historically been referred to as drug allergies, allergic drug hypersensitivity reactions, or drug hypersensitivity reactions (DHR) [1,4,5]. These events are also often categorized based on timing, being referred to as “immediate” (clinical signs occurring within six hours) and “delayed” (or “non-immediate”; clinical signs appearing >5 days after the first dose of drug course) [1,2,3,4,5]. Among these idiosyncratic drug events that involve the immune system, some are not antigen-specific (“pseudo-drug allergy”), and in immediate reactions, they are sometimes referred to as “non-allergic anaphylactic” or “anaphylactoid” reactions (see pathogenesis section for details). While this review will sometimes refer to these pseudo-allergic reactions, it will mainly focus on true antigen-specific drug reactions.

”Immediate” DHR are the manifestation of a type I hypersensitivity against the drug and are traditionally thought to be IgE-mediated (e.g., urticaria, anaphylaxis; see next section for details on pathomechanisms); the latter reactions are the manifestation of a type II, III, or IV hypersensitivity and are mediated via drug-specific IgG antibodies or drug-specific cytotoxic T lymphocytes (e.g., maculopapular eruptions, toxic epidermal necrolysis, hepatitis, immune-mediated hemolytic anemia) [1,3,4]. It is important to note, however, that DHR of type II, III, or IV can sometimes start after less than 5 days of exposure (potentially even within the first 24 h) in patients who were pre-sensitized during previous exposures. These cases being relatively uncommon, type I DHR reactions are usually called “immediate” and the others “delayed”, and this is how these terms will be used in this article. This review will focus on immune-mediated adverse drug reactions where the patient’s immune system targets a small drug or its metabolites (not a biological peptidic drug, nor a vaccine, nor a blood product).

The incidence of drug allergy in veterinary medicine has not been documented to date. However, the few case reports and retrospective studies on delayed DHR in small animals suggest an overall incidence (0.1%–3%) and clinical patterns similar to what is observed in humans [3,4]. Laboratory clinical tests (e.g., blood counts, biochemistry, and biopsy histology) and research assays (e.g., anti-drug and anti-tissue antibodies) conducted in dogs or cats with a history of such drug allergic reactions further suggest common underlying pathogenic mechanisms [4,6,7,8]. Drugs that are commonly associated with immediate DHR, in both human and veterinary patients, include antibiotics (βlactams, quinolones), neuromuscular relaxants, opioids, and NSAIDs. Beyond their significant incidence at the scale of the whole patient population, immediate DHR can also have a serious impact at the scale of the individual by their severity (as anaphylaxis can be life-threatening) and by the fact that they preclude from using the culprit drug again in this patient. Clinical signs of immediate DHR are those of type I hypersensitivity: they can affect the skin (e.g., urticaria), the skin-mucosal junction (angioedema, very common in dogs), the respiratory system (asthma-like reaction, very common in humans and cats), or the digestive system (e.g., acute diarrhea, common in dogs).

There are crucial first steps that the diagnosis of a DHR requires: taking a detailed history of the patient’s medical and pharmacological history; conducting a careful medical examination; and running some common blood tests (cell blood count, biochemistry) [1,2,3,4,5,9]. In addition, some in vivo tests are available to help confirm the reaction as well as the culprit drug: “dechallenge” (confirming that the clinical signs disappear on drug discontinuation); “drug provocation test” (DPT), the gold standard diagnostic step in DHR (“rechallenging” the patient with the suspected drug to reproduce the reaction); and skin testing (patch test or intradermal injection, administration of a small amount of suspected drugs on or in the skin to induce a localized inflammatory reaction). However, these in vivo tests can be unpractical (e.g., challenging the patient with all the suspected drugs in cases of polypharmacy) or even dangerous, as exposing the patient to the culprit drug (systemically or via the skin) could induce a new reaction that could be even more severe. This is why researchers and clinicians have spent decades trying to develop reliable diagnostic tools that would allow confirming DHR and/or identifying the culprit drug without exposing the patient to any risk [9,10,11]. These laboratory tests, with their specific strengths and weaknesses, are the focus of this review. Some of them have been used in veterinary patients where a type I hypersensitivity reaction to a non-drug stimulus was suspected, but not in cases of DHR. This review will also present their present place in small animal medicine.

## 2. Type I DHR Pathogenesis (Figure 1)

The characteristic clinical signs of drug-induced type I hypersensitivity reactions are the direct consequence of the cross-linking of cell membrane bound-IgE antibodies by the drug they target [1,5,12,13,14]. Anti-drug IgE antibodies are produced by drug-specific B lymphocytes that are regulated by drug-specific T-helper lymphocytes. These IgEs bind to the membranes of basophils (in blood or tissues) and mast cells (in tissues) that have a membrane rich in IgE Fc receptors (FcεR). The cross-linking activates the cells, leading to increased expression of certain membrane markers (e.g., CD203c) and degranulation. The degranulation itself induces the release of pre-formed and neosynthesized inflammatory and immune mediators (e.g., histamine and leukotrienes), as well as the addition of new membrane markers (e.g., CD63). The most important mediators released during degranulation are histamine and tryptase, but also leukotrienes and cytokines [12,14]. Several steps in this cascade of event are targeted by diagnostic tools, originally developed by researchers and eventually used by clinicians, after more or less validation for such application (see below).

It is important to note that some drugs can induce clinical signs similar to type I HS, but without the involvement of drug-specific IgE antibodies [4,14,15]. Such drug reactions are sometimes referred to as “anaphylactoid” (or “pseudo-allergic”) rather than “anaphylactic”, but a European task force on allergy preferred the term “non-allergic anaphylactic reaction” [16,17]. This is the case of certain opioids, NSAIDs, quinolones, or vancomycin, for instance. The mechanisms of these non-allergic reactions go beyond the scope of this review (e.g., direct pharmacological induction of histamine release; involvement of pathways such as the complement cascade or the MRGPRX2 receptor), but have been discussed elsewhere [12,15,18,19]. However, it is important to remember that immediate reactions to drugs such as opioids, NSAIDs, or quinolones might involve different pathomechanisms than IgE-mediated reactions to other drugs. This could explain why the use of the tests described below has often been disappointing in immediate reactions to opioids, NSAIDs, or quinolones.

## 3. Humoral In Vitro Tests

These tests measure a biomarker secreted in the patient’s blood during the reaction. There are two categories of humoral markers commonly measured in immediate DHR: (1) histamine and tryptase, which are not drug-specific and are markers of type I hypersensitivity in general; and (2) drug-specific IgE that identify or confirm which drug was associated with the immune reaction in cases of drug DHR. Note that secretory markers, such as histamine and tryptase, can also be measured ex vivo in the context of cellular assays (see Section 4).

### 3.1. Serum Histamine Concentrations

Histamine is mainly synthesized by basophils and mast cells, which release it extracellularly via degranulation upon activation. It is a key mediator of type I hypersensitivity [20,21].

Histamine levels can be measured using commercial immunoassays. Histamine is secreted within minutes of the anaphylactic reaction, but has a very short half-life as it is quickly metabolized after release [20,21,22]. Thus, samples should be collected within 15–20 min of the reaction onset, kept refrigerated, processed as quickly as possible, and should not be hemolyzed [20,23,24,25]. Urinary levels of histamine metabolites have been used as more stable surrogate markers for up to 24 h (and sometimes longer) after the reaction started [20,21,26]. Because of significant inter- and intra- individual variability, levels measured during a reaction should also be compared to baseline levels for accurate interpretation (pre- and/or post-reaction). A sensitivity ranging from 61 to 92% and a specificity ranging from 51% to 91% have been reported for plasma histamine tests in anaphylaxis diagnosis [9].

Circulating histamine levels tend to correlate with the severity of the anaphylactic reaction and are more likely to be increased than tryptase levels, especially in less severe cases (see below) [5,23,25].

#### Application in Veterinary Medicine

Various veterinary studies have measured histamine levels in canine plasma/serum using immunoassays originally developed for human samples (e.g., opioid-induced anaphylactoid reactions; cardiovascular injuries; heartworm infections; mast cell tumors) [27,28,29,30]. To the best of our knowledge, serum histamine concentrations have never been reported in naturally occurring cases of anaphalytic reaction, to a drug or other stimuli. We found a few studies of experimental anaphylactoid and anaphylactic reactions to drugs in research dogs that included histamine levels [31,32]. Because of the specific experimental methods used in these studies, however, it is difficult to extract clinically relevant data. Levels of histamine metabolites have also been reported in a few studies in dogs, including in a few experimental anaphylaxis projects [33], but not in a context of clinical anaphylaxis [34]. There is even less literature about serum histamine concentrations in cats [35]. It is possible that research has been limited in veterinary patients because clinicians have not felt the need for a laboratory test to help them diagnose an anaphylactic reaction or detect it earlier, before clinical signs become more obvious.

### 3.2. Serum Tryptase Concentrations

Serum tryptase is mainly produced by mast cells and to a much lower extent by basophils. The immature isoforms, α/β-protryptases, are produced continuously at low levels, proportionally to mast cell numbers. Mature β-tryptase is only released upon mast cell activation. Therefore, the mature form is a better indicator of the mast cells’ activational state and thus a better marker for anaphylactic reactions [36,37,38].

Tryptase levels are quantified using immunoassays. Relatively straightforward kits are commercially available, but presently only to measure total tryptase levels in human serum. However, the relatively short half-life of tryptase in serum significantly decreases its levels after 2 h, and it is advised that a blood sample within 1–3 hours is collected after the onset of clinical signs [38,39,40]. Levels of total tryptase >11.4 µg/L have been considered positive for a diagnostic of anaphylaxis in human patients [41]. However, several studies have shown that values below these thresholds have been observed in otherwise confirmed cases of anaphylaxis [41]. Interestingly, tryptase levels appear to correlate with clinical severity (e.g., decrease in blood pressure) [24,25,42,43]. Some authors have suggested that serial measurements can be more accurate [42,44]; tracking their citations for this comment led to an empirical study on an insect sting allergy where tryptase levels were measured at baseline, 15 min, and 60 min post-challenge [45]. Other authors have recently proposed an equation based on “reaction levels” (0.5–4 h after reaction onset) and “recovery levels” (≈24 h after recovery): tryptase levels were considered positive for a diagnosis of anaphylactic reaction if the reaction levels were > “2 + 1.2 × recovery levels” [46]. In type I hypersensitivity in general, serum tryptase levels seem influenced by the route of allergen exposure and the nature of the antigen [24,25].

Serum histamine and tryptase concentrations are the only tests commonly used in human medicine to confirm an anaphylactic reaction. However, because of high variability, potentially low specificity, and practical limitations in measuring plasma histamine levels, tryptase measurements are now favored when diagnosing anaphylaxis [47,48]. However, some authors consider that both tests display relatively low sensitivity, and careful history taking and clinical examining need to precede them [25,43]. It is important to note that the interpretation of histamine and tryptase levels might be challenging in patients with pre-existing conditions associated with abnormal mast cell activation (e.g., mastocytosis), as their baseline histamine and tryptase levels will likely be high whether or not an immediate DHR occurred.

#### Application in Veterinary Medicine

To the best of our knowledge, serum tryptase levels in dogs or cats with anaphylactic reactions have not been investigated to date. However, some researchers have used human reagents in order to quantify canine tryptase levels [49,50], but without reporting validation data in this species. Studies including feline tryptase levels are even more seldom and typically focused on intracellular detection, rather than circulating levels, in contexts different from type I hypersensitivity [51,52].

### 3.3. Circulating IgE Levels

#### 3.3.1. Total IgE

Total IgE concentrations are not diagnostically useful on their own in type I hypersensitivity reactions for a number of reasons. Contrary to the general perception, total IgE levels do not seem to be increased in all type I hypersensitivity reactions. Additionally, total IgE levels can be increased in numerous other clinical situations. Thus, total IgE levels cannot be easily interpreted when baseline values are not available, especially in patients suffering from other pre-existing IgE-mediated illnesses. Even when baseline values are available for comparison, increased levels do not simplify a large list of etiological options. Nevertheless, total IgE levels might be useful in diagnosing immediate DHR when used to normalize drug-specific IgE levels (see below for further details).

#### 3.3.2. Drug-Specific IgE

It is important to note that this assay is the only humoral test that is drug-specific. Indeed, those mentioned previously only indicate that an anaphylactic reaction took place without any information on its etiology in general or the culprit drug if it were drug-induced.

In these assays, the suspect drug is covalently bound to a carrier peptide that itself can be bound to a solid support (e.g., ELISA plate); this drug-containing solid phase is then exposed to the patient’s serum; if anti-drug IgEs are present they will bind to the drug; after washing away any unbound component of the serum, a species-specific anti-IgE antibody is added that will bind to any IgE that is bound to their drug target on the solid phase; these anti-IgE antibodies are subsequently detected thanks to their label, such as a radioactive (uncommon nowadays), fluorescent, or colored marker [9,10,41,53,54,55]. Immunoglobulins are much more stable than histamine and tryptase and their half-life is much longer. Thus, samples can be collected up to several years after the reactions, and can be frozen before testing. However, drug-specific IgE levels have been shown to decrease relatively quickly in some patients, requiring that the test be run sooner rather than later after the reaction [9,41,56]. A position paper prepared by an ENDA/EAACI Drug Allergy Interest Group recently stated that drug-specific IgE tests should therefore be conducted within three years after the DHR [9]. The statement only referenced a single study that only included 41 patients with a history of immediate allergic reaction to amoxicillin [56]. Thus, some authors disagree with this time limit [41,57]. Future guidelines about the appropriate timing of drug-specific IgE testing will be more beneficial if they are validated separately for individual drugs of concern, and, if they are based on more, and ideally larger, studies.

Drug-specific IgE tests have only been developed for a limited number of drugs and even fewer have been fully validated for clinical usage in human medicine [14,41,58]. Indeed, the development and validation is often complicated, or even impossible, largely because of the difficulty of obtaining specific positive controls (synthetic anti-drug IgE or patient serum proved to contain them). Yet, these tests are commonly used in human medicine in cases of immediate drug allergy to some β-lactams (probably the most commonly used IgE tests), muscle relaxant drugs, and opioids [14,41,59]. Note that, for the latter drugs, clinical reactions are usually non-allergic (“anaphylactoid”) in nature rather than IgE-mediated reactions. As for the “drug specificity” of certain anti-drug IgE and their detection assays, it is important to note that certain anti-muscle relaxant IgE appear to cross-react with certain opioids, antibiotics, and numerous other chemicals [60,61,62]. This is thought to be due to some substituted ammonium ions in the structure of these different compounds.

Drug-specific IgE tests show variable specificity and sensitivity, based on the tested drug (see detailed values in referenced reviews) [9,41,54]. Relatively low sensitivity of drug-specific IgE tests might be explained by various factors: (1) a poor selection of patients based on incomplete and/or inaccurate medical history and examination; (2) the fact that non-IgE-mediated immediate drug reactions (“anaphylactoid”) share a similar clinical pattern with true immediate allergic DHR; (3) drug binding to the assay matrix instead of the target antibody; (4) the use of a parent drug when the IgE target is its metabolite; (5) some hapten modification when binding the drug to the peptide carrier; or (6) high levels of total IgE. This last factor has been recently proposed as a significant issue when measuring drug-specific IgE [14,41,63,64,65,66]. Clinicians should therefore be aware that high total IgE levels can interfere with drug-specific IgE assays, and should work on “drug-specific IgE: total IgE ratios” whenever possible [65,66]. The use of “antigen-specific IgE: total IgE ratios” was originally developed in the field of human atopy (allergic asthma and atopic dermatitis) and is thought to have diagnostic, but also clinical, relevance [12,67,68]. Further work will be required to develop clinically relevant guidelines of drug-specific: total IgE ratios in human immediate drug allergy.

The specificity of drug-specific IgE tests (as their capacity to give a negative result for a non-allergic patient, not their “drug specificity”) appeared to be relatively high, but more recent evidence shows that it might be lower than originally thought [9,41]. Indeed, anti-drug IgE antibodies have been detected in patients who had received the drug, sometimes multiple times, in the absence of any adverse reaction [41,58]. Drug-specific IgE tests, like all the other assays described in this review, should therefore not be conducted to predict whether a patient is at risk to develop an immediate drug allergy reaction, but instead to confirm a reaction and/or the culprit drug in cases of polypharmacy. In any circumstances, drug-specific IgE results should be interpreted with caution, based on clinical history and examination, total IgE levels, sample collection timing, and other laboratory results (e.g., skin testing). Additionally, antigen-specific IgE circulating levels do not always accurately correlate with clinical sign severity [53,68,69]. Thus, the usefulness of drug-specific IgE levels as a diagnostic test relies heavily on a careful selection of patients based on medical history and clinical pattern, as well as on a careful validation of the drug-specific test itself.

#### 3.3.3. Application in Veterinary Medicine

Readers are referred to two relatively recent reviews that focus on IgE in dogs and cats, respectively, for additional details on this biomarker [69,70]. Kits to detect and quantify feline and canine IgE are now commercially available. Like in human medicine, total IgE levels have not proven useful in the diagnosis of non-drug related type I hypersensitivity in veterinary medicine and, to the best of our knowledge, have not been reported in cases of type I DHR in veterinary patients.

In veterinary medicine, research on antigen-specific IgE has mainly been focused on food allergy [71], fleabite hypersensitivity [72,73], allergic respiratory diseases [74,75,76], and atopic dermatitis [53,77,78]. Some private veterinary laboratories propose such allergen-specific IgE testing for clinical application. While drug-specific IgE testing has never been reported in any veterinary case of type I DHR, it is important to be aware of its weaknesses in veterinary atopy or food allergy diagnosis. They have been discussed in detail elsewhere, but some of their main shortcomings in veterinary medicine are the presence of allergen-specific IgE in healthy animals, a lack of inter-laboratory standardization, a lack of validation overseen by a regulatory agency, and a lack of patients with a certain diagnosis that could serve as reliable positive controls [53,79,80].

### 3.4. Other Humoral Markers Investigated in Type I Hypersensitivity Research

#### 3.4.1. Other Degranulation Enzymes

Carboxypeptidase and chymase are other enzymes secreted during degranulation. Less literature has been published on their use as diagnostic markers of anaphylaxis [81]. They are not secreted by basophils, but by tissue mast cells, so their use as serum biomarkers requires further investigation.

#### 3.4.2. Cytokine Profiles

Research on type I hypersensitivity has included a substantial amount of work on circulating cytokine profiles and their potential use in the diagnosis of anaphylactic reactions [32,82,83]. However, the complexity of their profile and the fact that they are involved in numerous other pathological processes will likely render cytokine levels/profiles more useful for prognosis and monitoring purposes than in diagnosis.

#### 3.4.3. Leukotrienes

Leukotrienes, such as LTC4, are produced during mast cell and basophil activation, and secreted during their degranulation [12,14]. Some researchers have investigated their potential as anaphylaxis biomarkers [84,85]. However, like cytokines, leukotrienes are produced by numerous types of cells and in numerous circumstances, so their use in the diagnosis of anaphylactic reactions in general and immediate DHR specifically will require further research.

#### 3.4.5. Platelet Activation Factor Levels

Some recent studies have considered the usefulness of platelet activating factor levels in the diagnosis of anaphylaxis with promising results, especially with the perspective of correlating these levels with clinical severity [86].

#### 3.4.6. Liver Injury Biomarkers

A relatively recent study on 40 dogs with mild allergic reactions and 61 dogs with severe anaphylaxis showed that increased gallbladder wall thickness and serum ALT activity were reliable early markers of severe anaphylactic reactions in dogs, with the latter test being easily conducted in most clinical settings [87]. The authors reported sensitivities of 93% and 85%, respectively, and specificities of 98% in both tests.

## 4. Cellular in Vitro Tests

As their name indicates, these tests focus on cells, but they can either detect the cells themselves or measure certain markers expressed on the cell surface or secreted by the cell. Unlike techniques relying on humoral markers, tests involving live cells require samples to be processed quickly and carefully because of the potential significant cell loss [88]. This is one of the pitfalls of in vitro cellular assays when considering their application in a clinical setting. On the other hand, a strong advantage presented by all cellular assays is that they also allow confirming the culprit drug in cases of polypharmacy.

In immediate drug allergy, the main cells involved in the pathogenesis are B lymphocytes that produce allergen-specific IgE, as well as circulating basophils and tissue mast cells which are both activated when their surface drug-specific IgE are cross-linked by the drug [1,5,12,13,14]. Lymphocytes and basophils circulate in the blood and are therefore more available for ex vivo testing than mast cells, especially for clinical applications.

### 4.1. Basophil-Related Tests

In human medicine, these tests measure the activation of the patient’s basophils when exposed ex vivo to the suspected drug. Thus, it is advised to wait at least two weeks after the reaction before attempting these tests so that collected basophils are not pre-activated [89]. On the other hand, the sensitivity of these tests can decrease significantly with time and it is advised to conduct them within a year of the reaction whenever possible [9,89,90]. It is important to note that, although each of the following tests use basophils, details of their protocols, such as effective ex vivo drug concentrations, might differ significantly [91]. For all the following assays, blood samples need to be collected and handled properly (e.g., EDTA or citrated tubes, refrigerated), and processed within a few hours [12,88,91,92].

#### 4.1.1. Measurement of Basophil Secretions

Upon IgE-induced degranulation, basophils will also release in vitro preformed histamine, measured by the “histamine release test” (HRT), and neosynthesized leukotrienes, quantified by the cellular antigen stimulation test (CAST) [9,89,93]. In this case, these secretions are not “humoral” markers per se, as they were not secreted in the patient’s blood. Both tests seem to have limited diagnostic value because of their usually poor sensitivity and specificity in human patients. This is why certain specialists do not recommend their use in drug allergy diagnosis in human medicine [9]. Furthermore, CAST (like the BAT, see below) appears to be useful in non-IgE-mediated reactions (“anaphylactoid”), such as those commonly induced by NSAIDs or opioids [89]. More recently, a flow cytometric approach to ex vivo histamine release by basophils (at a single cell level) has been developed, with several studies conducted successfully in cases of immediate DHR (HistaFlow®; e.g., neuromuscular blocking agents, βlactams) [94,95,96].

#### 4.1.2. Quantification of Basophil Membrane Markers

In human medicine, the most commonly used allergy test using basophils is the basophil activation test (BAT) that measures the increase in expression of cell membrane markers (mainly CD63 or CD203c) using flow cytometry [54,90,91,92,97]. CD63 is a basophil receptor expressed on granule membranes, which can be detected at high levels on the cell membrane upon the exocytosis of histamine-containing granules. CD203 is a cell membrane receptor expressed at low levels on basophils, but its expression is significantly upregulated when basophils are activated, with or without histamine release by degranulation [98,99].

The BAT has been shown to be a reliable tool in diagnosing certain DHR in human medicine. Variable sensitivities and specificities have been reported, ranging from 22% to 92% and from 40% to 100% (but usually >80%), respectively, with specificities overall higher than sensitivities [9,54,89,90,91,97,100]. Examples of such drugs are βlactams, quinolones, NSAIDs, muscle relaxant drugs, and pyrazolones. Note that, in the case of non-allergic immediate drug reactions (e.g., NSAIDs, opioids), the BAT can work in the absence of drug-specific IgE [89].

These assays were originally developed in the field of allergies to protein allergens, and were then adapted for immediate allergic reactions to non-peptide drugs. Commercial kits have been developed, but detailed protocols for use of them (e.g., drug concentrations tested) often vary between laboratories [9]. While the principles behind BATs are straightforward, the use of multiple negative and positive controls as well as consistent cytometer settings are required in order to limit the risk of an erroneous interpretation [12,54,91,101]. Interestingly, basophils from 5%–20% of human patients do not respond to BAT positive controls ex vivo, preventing the use of these assays for diagnosis purposes [9,12,14]. In addition, it is advised to test a large range of drug concentrations (more than 10,000-fold, if possible) [12,54]. Because circulating drug-specific IgE levels decrease over time in patients (see IgE section), the BAT sensitivity decreases accordingly when the basophil activation is based on drug-IgE cross-linking (i.e., the patient had a true drug allergic reaction, not an “anaphylactoid” one). It is therefore recommended that negative results be interpreted carefully three years post-reaction [9,56,90].

#### 4.1.3. Other Basophil-Based Tests

More recently, researchers have successfully explored the possibility of testing basophil activation via markers of intracellular signaling, such as the phosphorylation of certain signaling enzymes and transcription factors (e.g., p38 MAPK and STAT5) [63,102,103]. To the best of our knowledge, these recent technical advancements have not been applied to drug allergy cases yet.

#### 4.1.4. Applications in Veterinary Medicine

Basophil-based tests have not been used in the context of drug allergy in veterinary medicine to date. However, some researchers have adapted these assays to diagnose non-drug related allergies in companion animals. Some showed that plasma from dogs or cats with a history of atopy or food allergy can induce histamine release from human basophils in vitro [104,105,106]. Others used the patient’s basophils themselves, as done in human medicine [73,107]. While this review focuses on small animal medicine, the readers should be aware that the BAT has also been investigated for its research and clinical applications in horses with insect bite allergy (“summer eczema”) [108,109,110,111]. Note that, to date, this approach in dogs and cats has mainly focused on histamine release as readout rather than membrane receptor quantification by flow cytometry.

### 4.2. Drug-Specific Lymphocyte Detection

#### 4.2.1. Lymphocyte Transformation Test (LTT)

LTT is the gold standard in vitro assay to confirm a “delayed” DHR and/or identify which drug was involved in such a reaction [112,113,114,115]. The LTT detects the presence of circulating drug-specific lymphocytes in a patient’s blood. Such cells are also involved in the pathogenesis of immediate DHR: drug-specific IgE secreting B lymphocytes and T helper cells controlling the events [5,116]. Yet, it is important to note that the LTT is seldom used in immediate DHR [116,117]. A more detailed discussion of this assay will be conducted in our follow-up review on diagnostic tools for delayed DHR.

#### 4.2.2. Application in Veterinary Medicine

The LTT has been used in veterinary research for several decades [117]. Its use in the context of DHR has been very limited and to the best of our knowledge has never been reported in a case of immediate drug allergy in a veterinary patient. However, it can be found in other veterinary studies: e.g., anti-pathogen lymphocyte responses [118], vaccines [119], or in a few non-drug related IgE-mediated allergies in companion animals, such as cats with atopic rhinitis [75].

## 5. Genetic In Vitro Tests

Limited work has been published on the pharmacogenomics of immediate drug allergy in human medicine [5]. To date, most of the research in genetic predispositions in allergies has focused on non-drug related reactions [120]. Drug allergy pharmacogenetics has focused on “delayed” DHR; thus, a more detailed discussion on this subject will be found in our follow-up review about these drug reactions [121]. However, it appears that several genetic predispositions or even associations might be involved with IgE-mediated DHR: e.g., HLA, TNFα, IgE receptors, and certain cytokines [121,122]. The field of veterinary pharmacogenetics is relatively young and has not included any work on drug allergy so far [123].

## 6. Conclusions

Despite the weaknesses discussed in this review, it is crucial to remember that in vitro tests are safe and minimally invasive, which presently available in vivo tests are not. Historically, the first assays used in the diagnosis process of immediate DHR relied on humoral markers measured directly in patients’ serum. However, tests based on ex vivo measurements on patients’ cells (mainly basophils) have progressively taken the front row in the diagnosis of these adverse drugs reactions. It is important to remember that, to date, none of these assays, not even the most commonly used, has gone through a validation process with regulatory implications for clinical application in human immediate DHR yet (e.g., intra-laboratory and inter-laboratory reproducibility, or timing and condition guidelines for sample collection). This probably explains why no official consensus on which assays to use and how to conduct them emerges from exploring the literature on the subject of immediate DHR diagnosis. However, an expert group position paper was recently published to emphasize the importance of in vitro tests in the diagnosis of DHR and to highlight what the most reliable studies have shown so far [9]. To be valuable as a diagnostic tool in these drug reactions, a test would have to be conducted on samples taken soon after the onset of clinical signs (to manage the reaction itself that could be quickly life-threatening) and/or on samples collected once the patient will have recovered (to manage the patient pharmacologically in the future: e.g., identification of a drug not to be used again in this patient). Ideally, the test should identify or confirm the culprit drug in cases of polypharmacy. In any case, the most accurate diagnosis will likely rely on a panel of biomarkers rather than a single assay [25,43]. Any test validated for human patients will then have to be carefully evaluated in veterinary patients, which in itself will require further work in the neglected field of type I DHR in veterinary medicine.

## Figures and Tables

**Figure 1:**
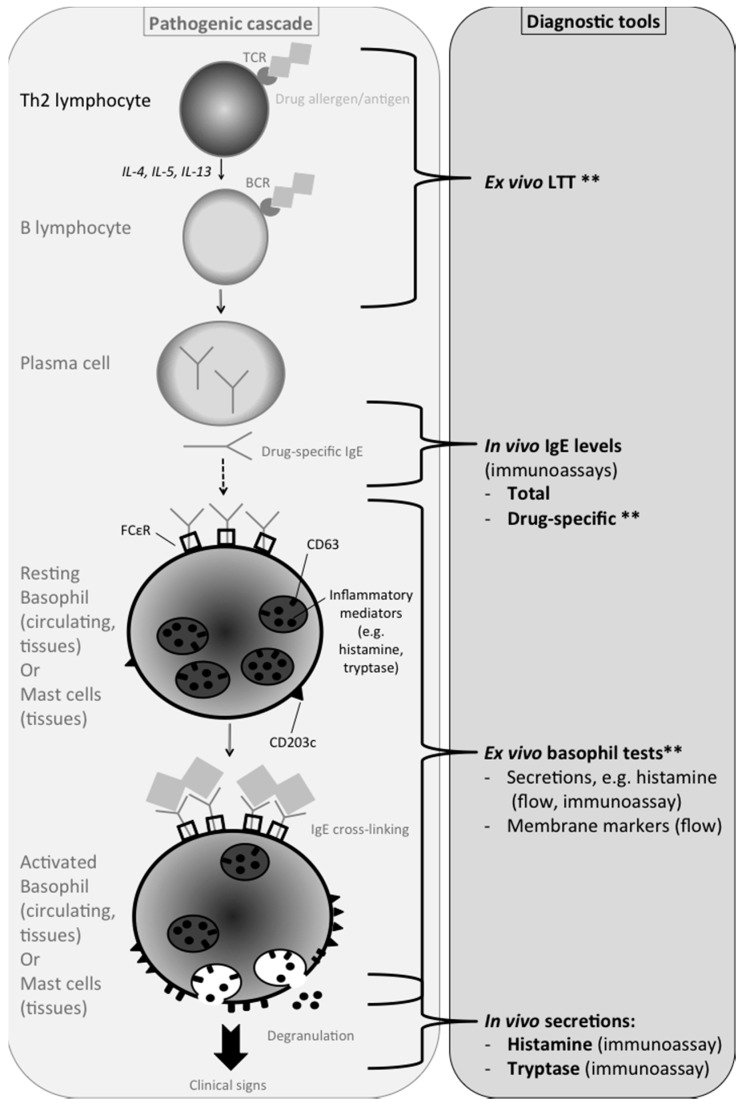
In vitro tests available based on the pathogenic events involved in immediate drug AHR. TCR = T cell receptor; BCR = B cell receptor; IgE = Immunoglobulin class E; FcεR = IgE receptor; LTT = Lymphocyte Transformation Test; ** tests that also confirm the nature of the culprit drug; “in vivo”: marker secreted in the patient during the reaction, but measured in vitro in a blood sample; “ex vivo”: a marker secreted in vitro by the patient’s cells.
