# Erratum: Lavergne, S.N. In Vitro Research Tools in the Field of Human Immediate Drug Hypersensitivity and Their Present Use in Small Animal Veterinary Medicine *Vet. Sci.* 2017, *4*, 1

**DOI:** 10.3390/vetsci5010006

**Published:** 2018-01-18

**Authors:** Sidonie N. Lavergne

**Affiliations:** Department of Veterinary Clinical Medicine, College of Veterinary Medicine, University of Illinois-Urbana-Champaign, 2001 South Lincoln Av, Urbana, IL 61802, USA; sidolavergne@hotmail.com

Due to an error during production, the author, Lavergne S. Lavergne’s name in the published paper [1] was incorrect. The author’s name should be changed to Sidonie N. Lavergne. 

We apologize for any inconvenience caused to the readers by this error. The change does not affect the scientific results. The article will be updated and the original will remain on the article webpage. 
